# Characteristic Gene Expression Profiles of Human Fibroblasts and Breast Cancer Cells in a Newly Developed Bilateral Coculture System

**DOI:** 10.1155/2015/960840

**Published:** 2015-06-11

**Authors:** Takayuki Ueno, Jun Utsumi, Masakazu Toi, Kazuharu Shimizu

**Affiliations:** ^1^Department of Breast Surgery, Kyoto University Hospital, Kyoto 606 8507, Japan; ^2^Department of Breast Surgery, Kyorin University Hospital, Tokyo 181 8611, Japan; ^3^Graduate School of Pharmaceutical Sciences, Kyoto University, Kyoto 606 8501, Japan; ^4^Cancer Institute, Japanese Foundation for Cancer Research, Tokyo 135 8550, Japan

## Abstract

The microenvironment of cancer cells has been implicated in cancer development and progression. Cancer-associated fibroblast constitutes a major stromal component of the microenvironment. To analyze interaction between cancer cells and fibroblasts, we have developed a new bilateral coculture system using a two-sided microporous collagen membrane. Human normal skin fibroblasts were cocultured with three different human breast cancer cell lines: MCF-7, SK-BR-3, and HCC1937. After coculture, mRNA was extracted separately from cancer cells and fibroblasts and applied to transcriptomic analysis with microarray. Top 500 commonly up- or downregulated genes were characterized by enrichment functional analysis using MetaCore Functional Analysis. Most of the genes upregulated in cancer cells were downregulated in fibroblasts while most of the genes downregulated in cancer cells were upregulated in fibroblasts, indicating that changing patterns of mRNA expression were reciprocal between cancer cells and fibroblasts. In coculture, breast cancer cells commonly increased genes related to mitotic response and TCA pathway while fibroblasts increased genes related to carbohydrate metabolism including glycolysis, glycogenesis, and glucose transport, indicating that fibroblasts support cancer cell proliferation by supplying energy sources. We propose that the bilateral coculture system using collagen membrane is useful to study interactions between cancer cells and stromal cells by mimicking in vivo tumor microenvironment.

## 1. Introduction

The microenvironment of cancer cells has been suggested to play critical roles in cancer development, progression, and therapeutic response. Cancer cells are supported by surrounding stromal cells such as fibroblasts, macrophages, myoblasts, and endothelial cells [1]. Fibroblasts that surround and interact with cancer cells have been called cancer-associated fibroblasts (CAFs) which can exert unique roles to support cancer cell growth [1]. These supporting effects via cell-cell cross talk may be different according to cancer cell types and characteristics, which remains to be elucidated.

To analyze cell-cell cross talk in vitro, several types of in vitro coculture systems such as a direct physical contact, an interaction coculture, and a transwell system have been developed [2–6]. In a direct contact, two types of cells are grown together in physical contact whereas, in an interaction coculture, two cell types are grown separated by a membrane and contact via soluble factors [7]. In a transwell system, one type of cells is grown on microporous membranes inserted in culture vessels where the other cell type is grown on the bottom and they communicate via soluble factors. These methods are useful to analyze cell-cell cross talk between tumor and nontumor cells in a single culture system. However, the organization of tumor and nontumor cells is different from in vivo conditions where tumor cells and stromal cells communicate through extracellular matrix such as collagen, neither through conditioned media nor by direct contact. Recently, a micropatterned coculture system has been introduced [8]. Epithelial cells are cultured on circular spots of extracellular matrix and subsequently stromal cells are seeded in the space between spots [8]. This system allows for an organized culture condition where epithelial cells on extracellular matrix are surrounded by stromal cells, which is similar to an in vivo condition. However, epithelial cells communicate with stromal cells through direct contact or soluble factors but not through extracellular matrix.

Here we have developed a novel bilateral coculture system in vitro to resemble in vivo conditions of cancer and stromal cells with extracellular matrix. By using three different subtypes of breast cancer cell lines and normal fibroblasts, we examined the interaction between cancer cells and fibroblasts and analyzed changes in gene expressions of both cancer cells and fibroblast to study a cross talk between cancer cells and stromal cells.

## 2. Materials and Methods

### 2.1. Cell Culture System

A suspending two-sided microporous collagen membrane with polystyrene reinforced outer frame (AteloCell, Koken Co. Ltd., Tokyo) was positioned in the culture medium in 50 mm diameter culture vessel. Normal human dermal fibroblasts (NHDF (NB) cells, Kurabo Industries Ltd., Osaka) were cocultured with one of the three different human breast cancer cell lines (luminal MCF-7, HER2-positive SK-BR-3, and triple-negative HCC1937) in this system. Normal human dermal fibroblasts were cultured on the lower side of collagen membrane (6 cm dish, 10% fetal bovine serum (FBS)/Dulbecco's modified essential medium (DMEM) for 1 day) and then breast cancer cells were inoculated and cultured on the upper side to form bilateral coculture (in 6 cm dish, 10% FBS/DMEM, 37°C for 3 days). For cross talk conditions, cancer cells (upper side) and fibroblasts (lower side) were cocultured in this system. For control conditions, the same cells were cultured on both sides of the bilateral membrane such as fibroblasts (upper side) and fibroblasts (lower side) or cancer cells (upper side) and cancer cell (lower side). Both sides of cells are able to interact through the collagen membrane and conditioned medium via secreted mediators.

### 2.2. Microscopic Observation

To confirm the condition of bilateral coculture with microscopic observation, collagen membrane after coculture for 3 days was collected and washed with phosphate buffered saline (PBS) (pH 7.4) twice and fixed with formalin/PBS and stained with haematoxylin and eosin. For electron microscopy, an ultrathin section from duplicated membrane specimen was produced by an ultramicrotome (DiATOME Ultra45°) and stained with uranyl acetate followed by aqueous lead citrate.

### 2.3. Transcriptomic Analysis

To analyze cell-cell interaction between two cell populations, each population must be separately collected after cellular cross talk. The primary aim of our coculture system is to harvest independently each side of cell population after coculture to study the cross talk between normal cell and cancer cell. After coculture for 3 days, cells were collected from the collagen membrane and applied to transcriptomic profile analysis of cultured cells. mRNA was extracted from cells with QIAzol Lysis Reagent (QIAGEN, Hilden). Harvested mRNA was applied to transcriptomic analysis with the highly sensitive microarray (3D-gene DNA tip, Toray Industries, Inc., Tokyo) [9] according to the manufacture's instruction for one-color analysis.

### 2.4. Bioinformatics Analysis

From the resulting list of expression genes, top 500 upregulated and downregulated genes were selected. These top 500 genes were applied to bioinformatics analysis to annotate gene function. Top 500 genes from upregulated and downregulated gene lists were applied to the web-based bioinformatics tool of MetaCore Functional Analysis (Thomson Reuter/GeneGo) on ontology, molecular pathway, and enrichment functional analyses to estimate canonical biological responses. The gene ontology (GO) analysis provides gene function and network of expressed gene through the cell-cell cross talk with breast cancer cells and fibroblasts.

## 3. Results

**Figure 1:**
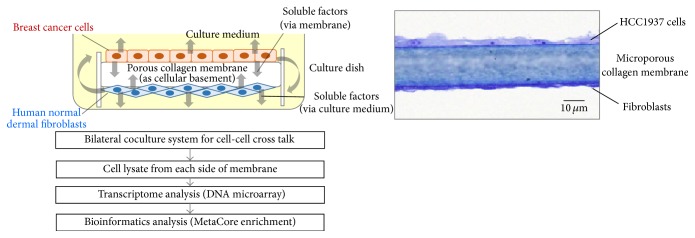
A concept view of bilateral coculture system and flow of experimental procedures. Breast cancer cells and fibroblasts were cocultured on each side of the bilateral microporous collagen matrix membrane. A suspending two-sided microporous collagen membrane with polystyrene reinforced outer frame was positioned in the culture medium in 50 mm diameter culture vessel. Cells were cultured as shown in the microscopic photo. Analytical flow is as illustrated by employing transcriptomic and bioinformatics analyses.

We have developed a bilateral coculture system to evaluate cell-cell cross talk by using collagen matrix membrane as shown in the experimental procedure in Figure 1. Two-sided collagen membrane was suspended by polystyrene reinforced outer frame in the culture medium. Breast cancer cells and fibroblasts were separately cultured on each side of the collagen membrane which played a role of extracellular matrix. The collagen membrane is composed of microporous matrix structure with the thickness of 20 micrometers and pores of less than 1-micrometer diameter to prevent cell migration into the membrane. The cancer cells and fibroblasts interact via soluble mediators through membrane and outer culture medium.

**Figure 2:**
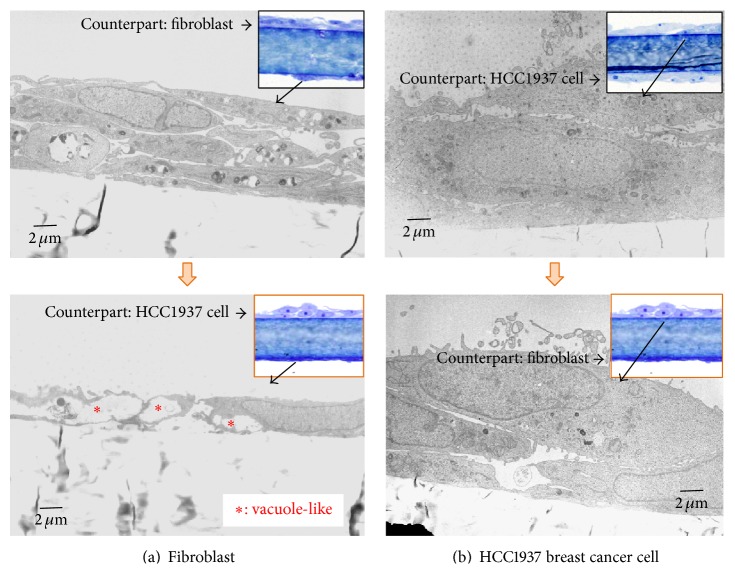
Electron microscopic views of cells in coculture. (a) Fibroblasts were cocultured with HCC1937 breast cancer cells (lower photo). As a control, fibroblasts were cultured with fibroblasts (upper photo). In coculture with HCC1937 cells, morphological change of cellular body shape and vacuole-like spaces were observed. (b) HCC1937 breast cancer cells were cocultured with fibroblasts (lower photo). As a control, HCC1937 cells were cultured with HCC1937 cells (upper photo). By coculture with fibroblasts, morphological changes of round shape were observed. Optical microscopic photos were shown at the top right corner.

We examined the morphological change of cocultured cells in this system by electron microscope. As shown in Figure 2, the cellular morphology and intracellular structures of cells in coculture were compared with those in the control condition. The control condition was the culture with the same cells on both sides, such as combinations of fibroblasts with fibroblasts or cancer cells with cancer cells. In the coculture of fibroblasts and cancer cells, HCC1937 cells showed a little round shape similar to that observed at mitotic phase, whereas fibroblasts showed a slim and waste shape with intracellular vacuole-like structures.

**Figure 3:**
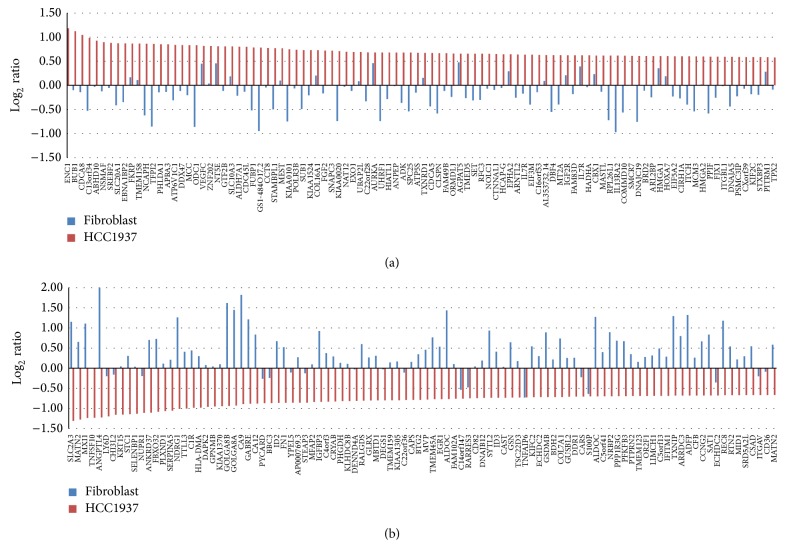
Alteration in individual gene expression in HCC1937 cells and fibroblasts in coculture. (a) Upregulated gene expression profile of mRNA in cocultured HCC1937 breast cancer cells and changes of corresponding genes in fibroblasts. (b) Downregulated gene expression profile of mRNA in cocultured HCC1937 breast cancer cells and changes of corresponding genes in fibroblasts.

After 3 days of coculture, cells were independently collected from each side of the membrane and mRNA was extracted and applied to transcriptomic analysis.  Figure 3(a) showed an upregulated gene expression profile of mRNA from cocultured HCC1937 breast cancer cells compared with control HCC 1937 cells (red bar) and alterations in expression of corresponding genes in cocultured fibroblasts compared with control fibroblast (blue bar). Interestingly, most of the genes upregulated in HCC1937 cancer cells were downregulated in fibroblasts and thus changing patterns of mRNA expression seemed to be reciprocal between HCC1937 cells and fibroblasts, suggesting that HCC1937 cells and counterpart fibroblasts exert distinct functions by interacting with each other. Among top 100 upregulated genes in HCC1937 cells, 77% of genes were downregulated in fibroblasts.

Figure 3(b) showed a downregulated gene expression profile of mRNA of cocultured HCC1937 breast cancer cells (red bar) with alterations in corresponding genes of cocultured fibroblasts (blue bar). Like upregulated genes, changes in most genes seemed to be reciprocal between HCC1937 cells and counterpart fibroblasts in the coculture condition. Among top 100 downregulated genes of HCC1937 cells, 82% of the genes were upregulated in fibroblasts.

Gene ontology analysis revealed that HCC1937 cells showed an increase in mitosis-associated genes and carbohydrate metabolic process (Table 1), suggesting that HCC1937 cells received proliferation stimulus through coculture with fibroblasts, which is consistent with the morphological changes observed in Figure 2. SKBR3 and MCF7 cells cocultured with fibroblasts also upregulated genes associated with cellular activity as shown in Table 1.

According to gene ontology analysis, HCC1937 cells showed a decrease in acute inflammatory response genes and phospholipid metabolic process (Table 2). SKBR3 and MCF7 cells cocultured with fibroblasts also downregulated genes associated with cellular transport as shown in Table 2.

Similar enrichment analyses were performed for fibroblasts cocultured with cancer cells (Tables 3 and 4). The analyses showed that genes associated with cell death regulation, stress, hypoxia, and carbohydrate metabolism were upregulated in fibroblasts. These results seemed to indicate that cocultured fibroblasts provided beneficial effects for cancer cells for survival and proliferation. In fibroblasts, genes associated with cell mitosis and cell membrane components synthetic pathways were downregulated (Table 4).

To understand general events in cancer cell-fibroblast cross talk, commonly changed genes were extracted among three types of breast cancer cells cocultured with fibroblasts. Similarly, commonly changed genes in fibroblasts cocultured with three different types of breast cancer cell lines were extracted. To mine the common genes either upregulated or downregulated in cocultured cells, top 500 genes were annotated. Tables 5(a) and 5(b) show gene ontology biological process and metabolic network with the specific gene names. As shown in Table 5(a), the prominent upregulation was observed in genes associated with cell cycle and cell division. Breast cancer cells also showed an increase in genes associated with carbohydrate metabolism, TCA, and amino acid metabolism, while functional process, phosphatides acid pathway, and glucuronic acid pathway were downregulated in three cancer cell lines (Table 5(b)).

The top 30 genes of altered expression in each of the three different cancer cell lines were listed in Table 5(c). No commonly upregulated genes among three cancer cell lines were observed. However, a transcriptional coactivator complex subunit, mediator complex subunit 13 (MED13), a regulator of cell proliferation, differentiation, and transformation, FBJ murine osteosarcoma viral oncogene homolog (FOS), and nuclear protein, transcriptional regulator, 1 (NUPR1) were commonly downregulated in top 30 genes.

The enrichment analysis also revealed that commonly upregulated genes in fibroblasts were associated with single-organism cellular process, carbohydrate transport, and amino acid transport as shown in Table 6(a). Furthermore, commonly downregulated genes in fibroblasts were genes associated with immune response regulation, cholesterol biosynthesis, and lipid metabolism (Table 6(b)).

## 4. Discussion

In the present study, we have developed a new bilateral coculture system to evaluate cell-cell cross talk by using collagen matrix membrane. The membrane is made of microporous collagen sheet with thickness of 20 micrometers and pore of less than 1-micrometer diameter. Cells do not penetrate into the membrane but interact with each other via secreted soluble factors such as metabolites, cytokines, and exosomes. One of the technical advantages of this system is the coculture of different cells on each side of the collagen membrane, which resembles in vivo conditions as extracellular matrix between cancer cells and stromal cells. In addition, this system enables retaining cellular polarity and, thus, stromal cells interact with “basal” sides of cancer cells through collagen, which is also in line with in vivo conditions. Even if cancer cells, especially poorly differentiated cancer cells, lose polarity, cancer cells communicate with stromal cells mostly via extracellular matrix, which can be mimicked by this system.

Our system consists of not only the culture system but also the following procedures and data analyses. Our total system is as follows: different cells were cultured on each side of the bilateral membrane and separately harvested followed by mRNA extraction, transcriptome, and bioinformatics analyses. To optimize these procedures, we chose the bilateral microporous collagen membrane but not polystyrene and polysulfone based membranes.

We found that, in fibroblasts cocultured with breast cancer cells, genes associated with carbohydrate metabolism including glycolysis, glycogenesis, and glucose transport were upregulated while, in cancer cells, genes associated with the tricarboxylic acid (TCA) cycle were upregulated. Our result is in agreement with the study by Fiaschi et al. showing that, through tumor-stromal interplay, cancer cells were reprogrammed toward aerobic metabolism while CAFs were reprogrammed toward a Warburg phenotype [10]. They suggested that cancer cells develop a dependence on lactate produced by CAFs for their growth, which is assumed to be an adaptation strategy to a low glucose environment [10]. Thus, it is conceivable that targeting not only cancer cells but also stromal cells is necessary for successful anticancer treatment, especially treatment regulating metabolic processes.

Altered expression of genes in hypoxic response and cancer invasion were observed in the present analysis. Hypoxia inducible factor 1, alpha subunit (HIF1A) was downregulated in common in cancer cells (Table 5(b)), while stromelysin-1 (matrix metallopeptidase 3), vascular endothelial growth factor A (VEGF-A), and neuropilin-1 were upregulated in common in fibroblasts (Table 6(a)). These responses suggested that CAFs play a supportive role of cancer cell invasion via tissue remodeling and neovascularization. In fibroblasts, genes associated with cell death regulation, stress, hypoxia, and carbohydrate metabolism were upregulated. On the other hand, genes associated with cell mitosis and cell membrane components synthetic pathways were downregulated in fibroblasts. These results suggest that CAFs play roles to support cancer cells in multiple ways for survival and proliferation.

There are several reports studying coculture with cancer cells and fibroblasts. In the study by Camp et al. where a direct coculture and a transwell system were applied, luminal type cancer cells behaved differently from basal-like cancer cells when cocultured with fibroblasts [7]. Luminal type cancer cells upregulated proliferation-related processes while basal-like cancer cells increased cellular migration in the coculture. Similarly, Rozenchan et al. showed, by using a transwell system, a basal-like cell line MDA-MB231 increased motility-associated genes [11]. In our system, genes associated with cell cycle or mitosis were commonly upregulated in breast cancer cell lines, which is in concordance with the previous reports. Interestingly, basal-like cell line HCC1937 increased genes associated with cellular division rather than migration in our system. Since rapid proliferation of cancer cells is a key feature of basal-like breast cancers, our system reflected an important aspect in “in vivo” conditions of basal-like breast cancer cells. We believe that the coculture system to better mimic in vivo conditions is of great value for analysis of interaction between cancer cells and stromal cells.

One possible application of our coculture system will be a drug screening. High-throughput drug screening is a key process for discovery and efficient development of new compounds for anticancer therapy [12, 13]. Screening with monoculture system has a limitation in that tumor-stromal interaction cannot be assessed although stromal components in tumor tissues play pivotal roles in response to anticancer agents. The bilateral coculture system in the present study would provide a useful system for such a purpose for drug development.

In conclusion, we developed a bilateral coculture system and showed that, in the coculture, breast cancer cells increased mitotic response and TCA pathway while fibroblasts increased carbohydrate metabolism including glycolysis, glycogenesis, and glucose transport, which is consistent with the notion that CAFs support cancer cell proliferation by providing energy sources. We propose that the bilateral coculture system using collagen membrane is useful to study interactions between cancer and stromal cells and would help effective drug screening by mimicking in vivo tumor microenvironment.

## Figures and Tables

**Table 1:** Enrichment analysis: upregulation in each cancer cell line.

#	Upregulation: GO processes in MCF7	*p* value	FDR
1	Cellular process	2.840*E* − 07	8.455*E* − 04
2	Protein localization to cell junction	1.219*E* − 06	1.814*E* − 03
3	Regulation of cardiac muscle cell apoptotic process	2.189*E* − 06	2.172*E* − 03

#	Upregulation: GO processes in SKBR3	*p* value	FDR

1	Cell cycle	1.192*E* − 09	3.578*E* − 06
2	Apoptotic process	5.228*E* − 09	4.644*E* − 06
3	Cellular metabolic process	6.045*E* − 09	4.644*E* − 06

#	Upregulation: GO processes in HCC1937	*p* value	FDR

1	Nuclear division	1.054*E* − 12	2.428*E* − 09
2	Organelle fission	2.879*E* − 12	3.317*E* − 09
3	Cell division	1.106*E* − 11	8.495*E* − 09

#	Upregulation: metabolic networks in MCF7	*p* value	FDR

1	Carbohydrate metabolism_TCA and tricarboxylic acids transport	6.670*E* − 03	3.936*E* − 01
2	Carbohydrate metabolism_propionate metabolism and transport	7.222*E* − 03	3.936*E* − 01
3	Vitamin, mediator, and cofactor metabolism_folic acid	1.949*E* − 02	4.310*E* − 01

#	Upregulation: metabolic networks in SKBR3	*p* value	FDR

1	1-Hexadecanoyl-glycerol_3-phosphate pathway	7.085*E* − 07	4.411*E* − 05
2	1-Linoleoyl-glycerol_3-phosphate pathway	1.357*E* − 06	4.411*E* − 05
3	1-Oleoyl-glycerol_3-phosphate pathway	1.030*E* − 05	2.231*E* − 04

#	Upregulation: metabolic networks in HCC1937	*p* value	FDR

1	Carbohydrate metabolism_pyruvate metabolism and transport	6.259*E* − 03	2.072*E* − 01
2	Lipid metabolism_triacylglycerol metabolism	1.271*E* − 02	2.072*E* − 01
3	N-Acetyl-D-galactosamine pathway	6.763*E* − 02	2.072*E* − 01

**Table 2:** Enrichment analysis: downregulation in each cancer cell line.

#	Downregulation: GO processes in MCF7	*p* value	FDR
1	Establishment of localization	2.679*E* − 05	2.740*E* − 02
2	Transport	3.991*E* − 05	2.740*E* − 02
3	Peptidylglycine modification	6.089*E* − 05	2.740*E* − 02

#	Downregulation: GO processes in SKBR3	*p* value	FDR

1	Regulation of apoptotic process	1.195*E* − 12	1.799*E* − 09
2	Regulation of programmed cell death	1.578*E* − 12	1.799*E* − 09
3	Response to mechanical stimulus	1.664*E* − 12	1.799*E* − 09

#	Downregulation: GO processes in HCC1937	*p* value	FDR

1	Acute inflammatory response	9.865*E* − 19	2.218*E* − 15
2	Response to metal ion	1.618*E* − 12	1.818*E* − 09
3	Response to inorganic substance	2.566*E* − 12	1.923*E* − 09

#	Downregulation: metabolic networks in MCF7	*p* value	FDR

1	Lysophosphatidylserine pathway	8.304*E* − 04	2.574*E* − 02
2	Carbohydrate metabolism_glycolysis, glycogenesis, and glucose transport	3.844*E* − 03	5.958*E* − 02
3	1,2-Didocosapentaenoyl-sn-glycerol_3-phosphate pathway	1.913*E* − 02	1.158*E* − 01

#	Downregulation: metabolic networks in SKBR3	*p* value	FDR

1	GalNAcbeta1-3Gal pathway	5.291*E* − 04	3.122*E* − 02
2	Pentose phosphate pathways and transport	1.247*E* − 03	3.677*E* − 02
3	(L)-Alanine pathways and transport	9.059*E* − 03	1.782*E* − 01

#	Downregulation: metabolic networks in HCC1937	*p* value	FDR

1	Phosphatidylethanolamine pathway	5.054*E* − 05	5.559*E* − 03
2	O-Hexanoyl-(L)-carnitine pathway	5.897*E* − 04	2.359*E* − 02
3	Myristoyl-L-carnitine pathway	6.435*E* − 04	2.359*E* − 02

**Table 3:** Enrichment analysis: upregulation in fibroblasts.

#	Upregulation: GO processes with MCF7	*p* value	FDR
1	Regulation of programmed cell death	1.104*E* − 15	3.326*E* − 12
2	Regulation of cell death	5.311*E* − 15	8.004*E* − 12
3	Regulation of apoptotic process	3.070*E* − 14	3.085*E* − 11

#	Upregulation: GO processes with SKBR3	*p* value	FDR

1	Cellular component organization	8.231*E* − 11	2.156*E* − 07
2	Cellular component organization or biogenesis	2.519*E* − 10	3.299*E* − 07
3	Response to stress	3.864*E* − 07	3.373*E* − 04

#	Upregulation: GO processes with HCC1937	*p* value	FDR

1	Single-organism cellular process	2.687*E* − 11	4.223*E* − 08
2	Single-organism process	2.848*E* − 11	4.223*E* − 08
3	Response to hypoxia	6.596*E* − 11	4.909*E* − 08

#	Upregulation: metabolic networks with MCF7	*p* value	FDR

1	(S)-Citrulline pathway	1.930*E* − 04	2.277*E* − 02
2	Glycine pathways and transport	1.052*E* − 03	6.208*E* − 02
3	L-Serine pathways and transport	1.889*E* − 03	7.432*E* − 02

#	Upregulation: metabolic networks with SKBR3	*p* value	FDR

1	Carbohydrate metabolism_glycolysis, glycogenesis, and glucose transport	1.938*E* − 06	4.456*E* − 05
2	Carbohydrate metabolism_fructose metabolism and transport	7.287*E* − 04	8.380*E* − 03
3	(L)-Alanine pathways and transport	1.295*E* − 02	7.443*E* − 02

#	Upregulation: metabolic networks with HCC1937	*p* value	FDR

1	Carbohydrate metabolism_glycolysis, glycogenesis, and glucose transport	4.800*E* − 08	2.112*E* − 06
2	Carbohydrate metabolism_fructose metabolism and transport	3.818*E* − 06	8.400*E* − 05
3	Carbohydrate metabolism_galactose metabolism and transport	4.798*E* − 05	7.037*E* − 04

**Table 4:** Enrichment analysis: downregulation in fibroblasts.

#	Downregulation: GO processes with MCF7	*p* value	FDR
1	Mitotic cell cycle	5.614*E* − 48	1.698*E* − 44
2	Cell cycle	7.592*E* − 44	1.148*E* − 40
3	Mitotic cell cycle process	3.015*E* − 42	3.040*E* − 39

#	Downregulation: GO processes with SKBR3	*p* value	FDR

1	Regulation of lipid metabolic process	1.373*E* − 13	5.965*E* − 10
2	Positive regulation of biological process	8.382*E* − 12	1.820*E* − 08
3	Regulation of protein metabolic process	1.294*E* − 11	1.874*E* − 08

#	Downregulation: GO processes with HCC1937	*p* value	FDR

1	Translation	2.041*E* − 09	1.903*E* − 06
2	Cellular macromolecular complex assembly	2.157*E* − 09	1.903*E* − 06
3	Nuclear-transcribed mRNA catabolic process	2.488*E* − 08	1.313*E* − 05

#	Downregulation: metabolic networks with MCF7	*p* value	FDR

1	2-Oleoyl-glycerol_3-phosphate pathway	2.569*E* − 04	1.079*E* − 02
2	Lysophosphatidic acid pathway	7.980*E* − 04	1.676*E* − 02
3	1-Hexadecanoyl-glycerol_3-phosphate pathway	1.298*E* − 03	1.818*E* − 02

#	Downregulation: metabolic networks with SKBR3	*p* value	FDR

1	N-Acyl-sphingosine phosphate pathway	3.589*E* − 08	2.441*E* − 06
2	1,2-Didocosapentaenoyl-sn-glycerol_3-phosphate pathway	1.119*E* − 04	2.836*E* − 03
3	1,2-Dioleoyl-sn-glycerol_3-phosphate pathway	1.373*E* − 04	2.836*E* − 03

#	Downregulation: metabolic networks with HCC1937	*p* value	FDR

1	Amino acid metabolism_lysine metabolism and transport	5.363*E* − 03	1.634*E* − 01
2	Carbohydrate metabolism_pyruvate metabolism and transport	1.318*E* − 02	1.634*E* − 01
3	Glucosylceramide pathways and transport	1.905*E* − 02	1.634*E* − 01

**(a):** 

#	Upregulation: GO processes in common of MCF7, SKBR3, and HCC1937 (104 genes)	*p* value	FDR
1	Cell cycle	1.002*E* − 22	2.262*E* − 19
	*D53, CDC18L (CDC6), ECT2, MCM7, Bard1, Thymidylate kinase, TTK, Rabkinesin-6, VRK1, HDAC1, TIPIN, CDC20, Histone deacetylase class I, DCC1, RFC4, ORC6L, CD2AP, EXO1, *and *BORIS *		

2	Mitotic cell cycle	8.624*E* − 22	9.732*E* − 19
	*D53, CDC18L (CDC6), MCM7, TTK, Rabkinesin-6, VRK1, HDAC1, TIPIN, CDC20, Histone deacetylase class I, DCC1, RFC4, ORC6L, CD2AP, C15orf23, HSP70, MAD2a, PBK, *and *TOP2 alpha *		

3	Cell cycle process	1.898*E* − 19	1.428*E* − 16
	*D53, CDC18L (CDC6), ECT2, MCM7, Bard1, TTK, Rabkinesin-6, VRK1, TIPIN, CDC20, Histone deacetylase class I, DCC1, RFC4, ORC6L, CD2AP, C15orf23, HSP70, MAD2a, PBK, *and *TOP2 alpha *		

4	Mitotic cell cycle process	4.502*E* − 18	2.540*E* − 15
	*D53, CDC18L (CDC6), MCM7, TTK, VRK1, TIPIN, CDC20, DCC1, ORC6L, CD2AP, C15orf23, HSP70, MAD2a, PBK, TOP2 alpha, Tubulin alpha, RRS1, Aurora-A, MSH2, CDK inhibitor 3, CKS1, *and *Tome-1 *		

5	Cell division	2.367*E* − 16	1.068*E* − 13
	*CDC18L (CDC6), ECT2, Rabkinesin-6, VRK1, TIPIN, CDC20, DCC1, CD2AP, EXO1, C15orf23, HSP70, MAD2a, PBK, TOP2 alpha, Tubulin alpha, RRS1, Aurora-A, MSH2, CKS1, Tome-1, *and *CCAR1 *		

#	Upregulation: metabolic networks in common of MCF7, SKBR3, and HCC1937 (104 genes)	*p* value	FDR

1	Carbohydrate metabolism_TCA and tricarboxylic acids transport	3.939*E* − 03	7.900*E* − 02
	*ODO2, SLC25A21, *and *SUCB1*		

2	Carbohydrate metabolism_propionate metabolism and transport	4.270*E* − 03	7.900*E* − 02
	*ACAT2, ACYP1, *and *SUCB1*		

3	Amino acid metabolism_lysine metabolism and transport	8.343*E* − 03	1.029*E* − 01
	*ODO2, ACAT2 *		

4	Phosphatidylcholine pathway	2.664*E* − 02	1.764*E* − 01
	*HSP70, COASY *		

5	1,2-Didocosapentaenoyl-sn-glycerol_3-phosphate pathway	3.305*E* − 02	1.764*E* − 01
	*AP3D1, Tubulin alpha *		

**(b):** 

#	Downregulation: GO processes in common of MCF7, SKBR3, and HCC1937 (105 genes)	*p* value	FDR

1	Negative regulation of vasoconstriction	1.343*E* − 06	1.091*E* − 03
	*HSPA1A, HSPA1B, HSP70, *and *HIF1A *		

2	Positive regulation of erythrocyte differentiation	1.876*E* − 06	1.143*E* − 03
	*HSPA1A, HSPA1B, ID2, HSP70, *and *HIF1A *		

3	Response to mechanical stimulus	2.787*E* − 06	1.358*E* − 03
	*ITGB1, EGR1, KV1.5, JunB, HSPA1A, HSPA1B, p70 S6 kinases, HSP70, c-Fos, ASNS, *and *HIF1A *		

4	Response to radiation	5.797*E* − 06	2.355*E* − 03
	*AKR1C4, ITGB1, EGR1, AKR1C1, Catalase, JunB, HSPA1A, HSPA1B, HSP70, PUMA, DEC1 (Stra13), USP47, c-Fos, ASNS, *and *HIF1A *		

5	Protein refolding	6.979*E* − 06	2.430*E* − 03
	*HSPA1A, HSPA1B, ST13 (Hip), *and *HSP70 *		

#	Downregulation: metabolic networks in common of MCF7, SKBR3, and HCC1937 (105 genes)	*p* value	FDR

1	Phosphatides acid pathway	1.438*E* − 03	1.093*E* − 01
	*PPAP2, LPP3 *		

2	D-Glucuronic acid pathway	3.958*E* − 03	1.504*E* − 01
	*AKR1C4, TBP, *and *c-Fos *		

3	Steroid metabolism_pregnenolone and progesterone metabolism	1.201*E* − 02	3.043*E* − 01
	*AKR1C4, HSD17B8, *and *AKR1C2 *		

4	2-Arachidonoylglycerol_3-phosphocholine pathway	2.337*E* − 02	4.434*E* − 01
	*Tissue kallikreins, Prostasin, *and *HIF1A *		

5	(L)-Leucine pathways and transport	5.754*E* − 02	4.434*E* − 01
	*p70 S6 kinase2, OSCP1 *		

**(c):** 

Upregulated genes			Downregulated genes		
MCF7	SKBR3	HCC1937	MCF7	SKBR3	HCC1937
IFIT1	PTGS1	BUB1	STK19	DUSP1	SCGB1A1
AP1B1	CTSZ	CDCA8	HMOX1	**FOS**	TF
OAT	CYP1B1	C13orf34	MEIS3	EGR1	C20orf114
ECT2	ALDH3B2	ABHD10	NMT1	SYCE1	LYNX1
EFNA1	SFRS16	NSMAF	CRK	**NUPR1**	CLCA2
TMEM189-UBE2V1	BPNT1	KIF2C	DIABLO	NR4A2	CASP14
AMH	SLC39A7	SREBF2	WDR34	ENDOD1	CP
NFE2L2	ATF1	SLC20A1	D2HGDH	NR4A1	EGLN3
PDPK1	A4GNT	EBNA1BP2	ATP1B1	OR14K1	C7orf29
HSPA8	BLZF1	NCAPH	TIMM50	ATF4	MATN2
ZNF117	TUBA3C	TFPI2	TMEM183A	IFI6	SERPINA3
GM2A	EXO1	PHLDA1	GDI1	BAK1	TNFSF10
NUF2	ST3GAL4	ATP6V1C1	TMEM168	ASNS	TMC4
ANP32A	CSNK2A1	DDX47	COX7B	SCNM1	ANGPTL4
ACYP1	CARHSP1	ODC1	AIFM2	TPM4	LY6D
RCN2	C13orf37	VEGFC	NUP188	MXD1	S100A8
STX3	TRAF4	GTF2B	DUSP22	ACTA1	CHI3L2
TSSC1	C4orf43	SLC10A3	MAK16	CTGF	KRT15
HEBP1	DCTPP1	ALDH7A1	TROAP	**MED13**	PSCA
FAM132A	CCAR1	CDC45L	C19orf46	ZNF783	SELENBP1
TMED10	BAX	FUBP1	**MED13**	CXorf23	**NUPR1**
KTN1	DSCC1	GS1-484O17.2	CLDN4	ADM	ANKRD37
ACTR6	YWHAG	CCT8	SLC25A1	SREBF2	CLDN8
AURKA	TEX261	MEST	ARF1	ID1	FBXO32
NGRN	S100A9	NCAPG	PHF2	ZG16	PLXND1
ILK	FAM10A5	MKI67	FFAR3	RGS16	SERPINA5
STAT1	FKBP10	KIAA0101	SCAF1	CLIC4	**FOS**
PWP1	ACO2	SUB1	C2orf76	MED12	NDRG1
PABPC3	TMSB4X	KIAA1524	MYOM2	JUN	CRIP2
SLBP	VWA5B1	FGF2	KCTD17	RNF126	WFDC2

**(a):** 

#	Upregulation: GO processes in common versus MCF7, SKBR3, and HCC1937 (130 genes)	*p* value	FDR
1	Single-organism cellular process	1.586*E* − 10	4.679*E* − 07
	*SMURF, FTS, ATF-4, AP-3 sigma subunits, SAT-1, Tenascin-C, GLSL, COUP-TFII, GCR-beta, SLIT2, SLFN5, TRUNDD(TNFRSF10D), MCT1 (SLC16A1), Neuropilin-1, RRN3, GLSK, SMAD6, FoxD1, *and *NIP3 *		

2	Response to organic cyclic compound	3.170*E* − 10	4.679*E* − 07
	*Tenascin-C, COUP-TFII, GCR-beta, SLIT2, MCT1 (SLC16A1), SMAD6, TIMP3, MKP-3, MKP-1, Lysyl oxidase, Adipophilin, COUP-TFI, Stromelysin-1, SLIT3, Stanniocalcin 2, *and *VEGF-A *		

3	Single-organism process	7.615*E* − 10	6.297*E* − 07
	*SMURF, FTS, MOXD1, ATF-4, AP-3 sigma subunits, SAT-1, Tenascin-C, GLSL, COUP-TFII, GCR-beta, SLIT2, SLFN5, TRUNDD(TNFRSF10D), MCT1 (SLC16A1), Neuropilin-1, RRN3, GLSK, SMAD6, *and *TIMP3 *		

4	Response to endogenous stimulus	8.532*E* − 10	6.297*E* − 07
	*SMURF, AP-3 sigma subunits, Tenascin-C, COUP-TFII, GCR-beta, SLIT2, SMAD6, TIMP3, PINCH, Connexin 43, SMURF2, GCR-alpha, PDGF-C, MKP-1, Lysyl oxidase, Stromelysin-1, SLIT3, *and *VEGF-A *		

5	Organic anion transport	2.208*E* − 09	1.304*E* − 06
	*SAT-1, GLSL, MCT1 (SLC16A1), GLSK, MCT4, CAT-1 (SLC7A1), Connexin 43, GLUT3, Adipophilin, SLC25A4, SLC38A1, Carbonic anhydrase XII, SLC38A2, SLC27A3, CAT-3, SLC7A5, *and *ANT *		

#	Upregulation: metabolic networks in common versus MCF7, SKBR3, and HCC1937 (130 genes)	*p* value	FDR

1	Carbohydrate metabolism_glycolysis, glycogenesis, and glucose transport	9.568*E* − 06	4.497*E* − 04
	*PFKP, GLUT3, ALDOC, ENO2, ALDOA, *and *ENO *		

2	Carbohydrate metabolism_sucrose metabolism and transport	4.596*E* − 05	7.525*E* − 04
	*COUP-TFII, GLUT3, COUP-TFI, Glycogen phosphorylase, *and *PYGL *		

3	(L)-Proline pathways and transport	4.803*E* − 05	7.525*E* − 04
	*CAT-1 (SLC7A1), Glycogen phosphorylase, SLC38A2, CAT-3, SLC7A5 *		

4	L-Serine pathways and transport	1.369*E* − 04	1.538*E* − 03
	*CAT-1 (SLC7A1), SLC38A1, SLC38A2, CAT-3, *and *SLC7A5 *		

5	(S)-Citrulline pathway	1.636*E* − 04	1.538*E* − 03
	*CAT-1 (SLC7A1), Glycogen phosphorylase, CAT-3, *and *SLC7A5 *		

**(b):** 

#	Downregulation: GO processes in common versus MCF7, SKBR3, and HCC1937 (107 genes)	*p* value	FDR

1	Response to organic substance	1.120*E* − 10	3.750*E* − 07
	*ERG1, IDI1, HSBP3, Ribonucleotide reductase, Galpha(s)-specific prostanoid GPCRs, MGMT, Cathepsin S, GREM2, MMP-13, SFRS3, BMP2, H-FABP, ACAT2, MGST3, IBP2, NNMT, CCL2, *and *DNAJA3 *		

2	Regulation of immune complex clearance by monocytes and macrophages	4.480*E* − 09	4.999*E* − 06
	*CCL2, CCL13, Galpha(q)-specific peptide GPCRs, Galpha(i)-specific peptide GPCRs *		

3	Positive regulation of immune complex clearance by monocytes and macrophages	4.480*E* − 09	4.999*E* − 06
	*CCL2, CCL13, Galpha(q)-specific peptide GPCRs, *and *Galpha(i)-specific peptide GPCRs *		

4	Response to endogenous stimulus	1.450*E* − 08	1.213*E* − 05
	*Ribonucleotide reductase, Galpha(s)-specific prostanoid GPCRs, MGMT, Cathepsin S, MMP-13, SFRS3, BMP2, H-FABP, MGST3, IBP2, NNMT, CCL2, CCL13, *and *Galpha(q)-specific peptide GPCRs *		

5	Response to acid	2.446*E* − 08	1.638*E* − 05
	*Galpha(s)-specific prostanoid GPCRs, MGMT, BMP2, H-FABP, ACAT2, IBP2, CCL2, CCL13, Galpha(q)-specific peptide GPCRs, Galpha(i)-specific peptide GPCRs, PGD2R, PEDF (serpinF1), INSIG1, *and *CD9 *		

#	Downregulation: metabolic networks in common versus MCF7, SKBR3, and HCC1937 (107 genes)	*p* value	FDR

1	Steroid metabolism_cholesterol biosynthesis	3.670*E* − 06	1.468*E* − 04
	*ERG1, IDI1, ACAT2, DHC24, MVD, *and *DHCR7 *		

2	GalNAcbeta1-3Gal pathway	5.291*E* − 04	1.058*E* − 02
	*Coagulation factor X, Galpha(q)-specific peptide GPCRs, Galpha(i)-specific peptide GPCRs, *and *CD13 *		

3	N-Acyl-sphingosine phosphate pathway	8.346*E* − 03	1.113*E* − 01
	*Galpha(q)-specific peptide GPCRs, PLAU (UPA), *and *MMP-1 *		

4	Lipid metabolism_n-6 polyunsaturated fatty acid biosynthesis	3.289*E* − 02	3.270*E* − 01
	*FADS2, FADS1 *		

5	Glucosylceramide pathways and transport	5.067*E* − 02	3.270*E* − 01
	*FADS2, FADS1 *		
